# Special Issue “Advances in Coagulation and Anticoagulation”

**DOI:** 10.3390/ijms26167802

**Published:** 2025-08-13

**Authors:** Vance G. Nielsen

**Affiliations:** Department of Anesthesiology, The University of Arizona College of Medicine, Tucson, AZ 85724, USA; vnielsen@anesth.arizona.edu

The advancement of the clinical care of patients afflicted with hemorrhagic or thrombotic disease relies heavily on ongoing molecular investigations into the mechanisms of coagulopathy. With the above in mind, this Special Issue invited the submission of all works concerning coagulation and anticoagulation that cover both molecular aspects and mechanisms, including clinical trials involving new procoagulant or anticoagulant agents, in addition to original descriptions of preclinical animal models that detail the pharmacokinetic and pharmacodynamic impacts of such agents. Toxic interactions of organic and inorganic molecules were also of interest, taking the form of in vitro and in vivo models. This call for papers was broad in scope, and I was delighted that a diverse response in terms of focus and content was achieved [1,2,3,4,5]. These works will be subsequently highlighted in detail.

This Special Issue contained two reviews [1,2], with one focusing on how metals modulate platelets, coagulation, and fibrinolysis [1] and the second serving as a comprehensive review of the structure, synthesis, and activity regulation of factor VII activating protease (FSAP) [2]. In the case of FSAP, Kwiakowska presented the first of what is to be a series of review articles concerning this protein [2]. The history of the discovery of FSAP, its genetic locus, and location within humans, mice, and rats are presented in detail, as is its molecular forms and structure [2]. The endogenous activators, the process of autoactivation, and inhibitors of FSAP are displayed in the figure and text comprehensively. The precise role of FSAP in thrombotic disease (e.g., stroke) and inflammatory disorders is a source of ongoing investigation, and the articles included in this collection are the beginning of what promises to be an informative series of reviews of FSAP in the years to come [2].

In the second review, Nielsen et al. collated works that provided clear evidence of modulation of platelet activity, coagulation, and fibrinolysis that spanned the molecular to clinical levels of evidence [1]. While the role of calcium (Ca) is well known within the processes that constitute hemostasis, there are twenty-four additional metals, from aluminum (Al) to zinc (Zn), that have been found to affect platelet activity, coagulation, and fibrinolysis [1]. Some metals are administered to patients as dietary supplements, some form part of diagnostic dyes, some are part of toxic environmental exposures in the environment in general, and some are a product of industrial waste [1]. Other metals can be found as a constituent of prodrugs or occasionally in actual or potential chemotherapeutic agents that contain platinum (Pt) or ruthenium (Ru) [1]. Using Ru as an example, in this review, it is noted that the simple molecule RuCl_3_ dissolved in phosphate buffer enhances prothrombin activation in human plasma; furthermore, the Ru-containing chemotherapeutic agent NAMI-A dissolved in water enhanced plasmatic coagulation as determined by means of thromboelastography [1]. As a follow-up investigation, it was determined that the Ru-containing compound CORM-2, which has been used in hundreds of in vitro and in vivo investigations due to its carbon monoxide-releasing characteristics, also forms a Ru radical species that enhances coagulation and inhibits fibrinolysis in human plasma [6]. It is hoped that the findings presented in this review will provide the readership with a comprehensive view of the metal-mediated modulation of hemostasis [1].

In two of the three prospective investigations included in this Special Issue, the authors utilized in vitro and in vivo animal models to ask and answer interesting questions concerning comparative hematology [3] and anticoagulant compounds derived from hematophagous organisms [4]. Liang et al. explored the thrombin-generating effects of human tissue factor (TF) and activated Factor XI (FXIa) in human plasma and the following animal plasmas: guinea pig, mouse, rat, rabbit, cow, sheep, and goat [3]. The authors used fluorescence produced by 7-amino-4-methylcoumartin to assess thrombin generation (TG) and fibrin clot formation (CF) as the tests to compare the effects of these human intrinsic (FXIa) and extrinsic (TF) proteins in these different animal plasmas in order to determine which animals may show the greatest similarity to humans in their response [3]. The rationale for this fascinating investigation was that the design and testing of anticoagulants targeting human proteins need to be used in preclinical models, which mandates the characterization of multiple animal plasmas to determine which animal plasma most closely matches human plasma in a test-specific fashion [3]. The results are fascinating and the work comprehensive, making this article an excellent reference for members of the readership working in this field.

In a similar vein, Kostromina et al. also used animal models to address the efficacy of unique anticoagulant agents; in this case, however, they developed an in vivo, multispecies panel of critical hematological assessments to accomplish this goal [4]. Using the administration of unfractionated heparin to activate endogenous antithrombin as a standard, the authors compared oral dabigatran etexilate (chemically related to the antithrombotic agent derived from the medicinal leech *Hirudo medicinalis*, known as hirudin-1) and intravenous variegin (derived from the tick *Amblyomma variegatum*). The authors used mice administered the medication of interest that were sacrificed at specific times for standard hematological assessments as a pharmacodynamic model. In turn, rats underwent either tail bleeding time tests or formed part of a standard inferior vena cava constriction model to produce thrombi that could be weighed post mortem. These three animal models provided insight into the efficacy of each antithrombotic agent in a unique fashion that is planned to be utilized by this group in the future with other hematophagous organisms as sources of novel and potentially clinically useful antithrombotic agents.

In the final work of this collection, contributed by Reda et al., the authors discovered a hematological molecular finger print of malignant disease associated with a rare thrombotic disorder [5]. Splanchnic vein thrombosis (SVT) has an incidence of one per 100,000 person/years, and its etiology can be complex, involving comorbidities such as hepatic cirrhosis; of note, myeloproliferative neoplasms (MPNs) are a major risk factor of SVT [5]. Using a comprehensive hematological panel of assessments, the authors found that elevated circulating thrombin and activated protein C (APC) in the presence of SVT was greatest when an MPN was present [5]. Thus, in the case of known MPNs, if circulating thrombin and APC levels are abnormally increased, then the patient may be hypercoagulable and at risk of thrombotic events. Conversely, if the patient presents with SVT and circulating thrombin and APC are observed, then a search for MPN should perhaps be pursued. While the number of patients in this study was small—not surprising given the incidence of the disease—the manuscript serves as a hypothesis-generating work to direct future investigations.

In summary, this Special Issue contains unique and diverse works that are expected to appeal to the interests of diverse groups in the molecular hematology field. It is our desire that the readership benefits from this body of knowledge.
